# Innate immune activation as cofactor in pemphigus disease manifestation

**DOI:** 10.3389/fimmu.2022.898819

**Published:** 2022-07-19

**Authors:** Ramona A. Eichkorn, Morna F. Schmidt, Elias Walter, Michael Hertl, Jens Malte Baron, Jens Waschke, Amir S. Yazdi

**Affiliations:** ^1^ Department of Dermatology, Eberhard Karl University of Tuebingen, Tuebingen, Germany; ^2^ Department of Dermatology, Rheinisch-Westfälische Technische Hochschule Aachen (RWTH) Aachen University, Aachen, Germany; ^3^ Department I, Institute of Anatomy and Cell Biology, Ludwig Maximilian University of Munich (LMU), Munich, Germany; ^4^ Department of Dermatology and Allergology, Philipps University of Marburg, Marburg, Germany

**Keywords:** interleukin 1, inflammasomes, caspases, desmoglein, pemphigus vulgaris

## Abstract

Molecular mechanisms underlying auto-antibody-induced acantholysis in pemphigus vulgaris are subject of current research to date. To decipher the discrepancy between ubiquitous antibody binding to the epidermal desmosomes, but discontinuous disease manifestation, we were able to identify Ultraviolet A (UVA) as a cofactor for acantholysis. UVA induces interleukin (IL)-1 secretion in keratinocytes, mirroring innate immune system activation. In an *in vitro* keratinocyte dissociation assay increased fragmentation was observed when UVA was added to anti-Desmoglein 3 Immunoglobulins (anti-Dsg3 IgG). These results were confirmed in skin explants where UVA enhanced anti-Dsg3-mediated loss of epidermal adhesion. The UVA-mediated effect was blocked *in vitro* by the pan-caspase-inhibitor zVAD-fmk. Thus, we introduce UVA as a caspase-dependent exogenous cofactor for acantholysis which suggests that local innate immune responses largely contribute to overt clinical blister formation upon autoantibody binding to epidermal cells in pemphigus vulgaris.

## Introduction

Pemphigus vulgaris (PV) is a chronic autoimmune blistering disease characterized by the production of autoantibodies against Desmogleins (Dsg) 3 and Dsg1 causing dissociation of keratinocytes in the cell complex (acantholysis) and subsequent blister formation of the mucous membranes and the skin (1–3). At present, the complex mechanisms causing acantholysis are not fully understood but are known to require signaling mechanisms besides direct inhibition of desmoglein binding (4). PV still poses a therapeutic challenge due to severe co-morbidities of current treatment options, a refractory course (5, 6) and a high mortality when untreated (7). Although antibodies bind ubiquitously to the epidermis, acantholytic blisters only occur at distinct areas of the skin and the mucous membranes (8). Exogenous cofactors activating the innate immune system might be a key element in explaining the discrepancy between acantholysis and a lack of blister formation. Accordingly, in blister fluid from PV patients, increased levels of innate cytokines were detected which suggests a pathogenic role of innate immune activation (9–11). There are hints that IL-1α can increase complement activation which is observed in a majority of PV patients and might play an important role in acantholysis (12). Additionally, the innate immune system can modulate T cell-mediated disorders such as psoriasis (13) and presumably also PV (14).

IL-1 is an inflammasome-related proinflammatory cytokine involved in auto-immune and auto-inflammatory processes [reviewed by (15)]. It connects innate and adaptive immune responses by orchestrating lymphocyte differentiation (16).

Innate contributors in the pathogenesis of blistering diseases belonging to the pemphigoid-group have been early voiced due to the rich lesional inflammatory infiltrate of neutrophils, eosinophils and lymphocytes (17). Bullous pemphigoid (BP) is proposed to be a T-cell dependent, mainly Th2-like autoimmune disease with production of pathogenic IgG antibodies against the BP180 ectodomain of hemidesmosomes (18). This was based on the discovery of increased Th2-related (19, 20) cytokines in BP patients. Increased levels of complement factors (21–23), innate cytokines IL-1α and IL-1β (24, 25), Th-type cytokines IL-3, IL-4, IL-6, IL-10, and Granulocyte-macrophage colony-stimulating factor were detected in the sera and/or blister fluids from BP-patients (26).

The interaction of the innate and adaptive immune system and the release of cytokines are poorly understood in PV. This study aims to dismantle the local impact of cytokines on cell-dissociation apart from their role as key mediators in cell-mediated processes of autoantibody production in PV. Altered T-cell subsets are known to fuel the immuno-pathogenesis and inflammation in the skin [reviewed by (27, 28)]. Related to this, extensive changes in the cytokine network of pemphigus patients were described [reviewed by (29)]. Previous studies provided initial evidence for a local role of innate cytokines: IL-1-deficient mice as well as tumor necrosis factor-alpha receptor-deficient mice showed decreased susceptibility to PV-related antibodies (10). In addition, IL-1 upregulated tissue-type plasminogen activator secretion in the spontaneously immortalized keratinocyte cell line HaCaT (30), which might also be involved in PV-related acantholysis (31).

To improve treatment options and to obtain a better understanding of PV, we investigated whether IL-1-inducing stimuli, such as UVA irradiation led to an activation of the innate immune system as a cofactor for blister formation in PV. Here, we were able for the first time to identify UVA as a caspase-dependent exogenous cofactor in the pathogenesis of PV.

## Augmentation of acantholysis *via* inflammatory caspases

### UVA induces protein secretion of innate cytokines

To determine the effect of UVA irradiation as a potential cofactor in PV, the spontaneously immortalized keratinocyte cell line HaCaT was irradiated with UVA at 5 J/cm² (the titrated intensity that resulted in the best cytokine response, while cell viability was not affected, **Supplementary Figure 1**). Afterwards, cells were treated with IgG purified from PV sera (PV-IgG) which contained high levels of anti-Dsg3 IgG. Subsequently, gene expression of proinflammatory cytokines *IL1A, IL1B, IL6* and *IL8* was determined *via* real-time PCR (rtPCR) and the secretion of respective cytokines was measured by Enzyme-linked Immunosorbent Assay (ELISA).

**Figure 1:**
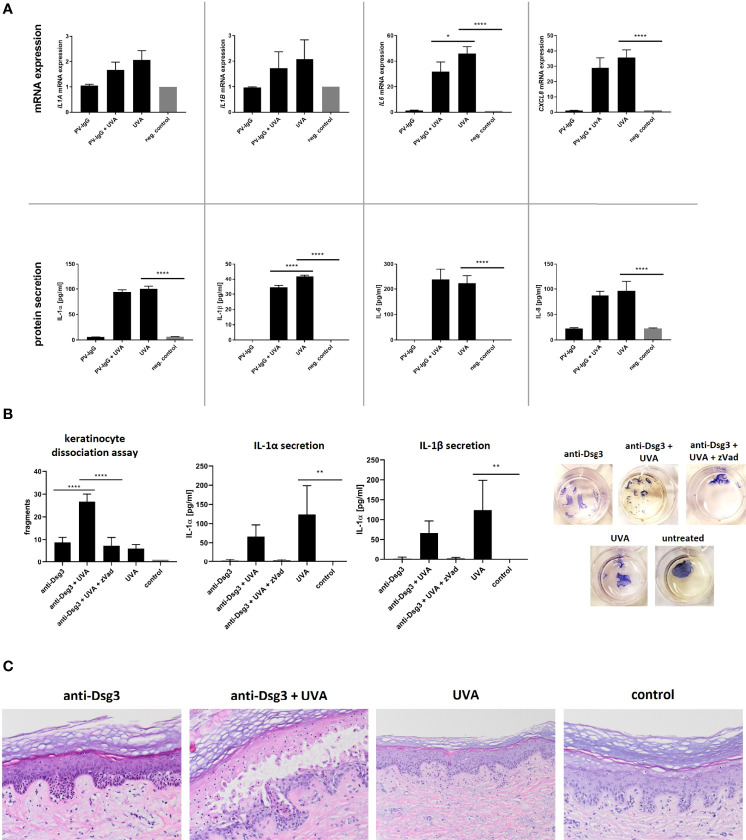
**(A)** HaCaT cells were irradiated with 5 J/cm^2^ UVA. Directly afterwards IgG purified from patient sera was added. After an incubation period of four hours, the expression of IL1A, IL1B, IL6 and IL8 was determined *via* rtPCR. Protein secretion was measured by ELISA. **(B)** After irradiation of HaCaT (5 J/cm^2^ UVA) the monoclonal anti-Dsg3 antibody AK23 was added for four hours. To block the effect caspase-activation zVad-fmk was added one hour prior to irradiation. A dispase-based keratinocyte dissociation assay was performed by applying shear stress on the epidermal monolayers. Cell fragments were stained with MTT and counted, IL-1 secretion was measured *via* ELISA. **(C)** Human skin explants were irradiated with 15 J/cm² UVA and incubated *ex vivo* for 48 hours with or without the monoclonal anti-Dsg3 antibody AK23 in increasing doses. Afterwards explants were HE stained. Error bars represent the SEM. *p < 0.05. The data evaluation was carried out *via* a One-way analysis of variance. **** p<0.0001; ** p<0.01; * p<0.05.

After treatment with UVA, HaCaT cells showed a two-fold increase in mRNA expression for *IL1A, IL1B* only but a robust induction of *IL6* and *IL8* compared to untreated cells (**Figure 1A**). The treatment with PV-IgG did not lead to any notable changes in the expression of the indicated cytokines which is in line with previous findings (32). Furthermore, UVA irradiation induced the secretion of IL-1α, IL-1β, IL-6 and IL-8. Stimulation with PV-IgG alone did not result in a significant increase in cytokine release (**Figure 1A**).

Combined stimulation with UVA followed by PV-IgG treatment showed no significant differences in cytokine gene expression and in protein secretion except for IL-1β compared to the stimulation with UVA alone.

### UVA enhances PV-IgG-induced acantholysis caspase-dependent *in vitro*

Next, we evaluated the pathogenicity of anti-Dsg3 antibody and UVA as a pro-inflammatory cofactor *in vitro*. Therefore, we applied a well-established dispase-based keratinocyte dissociation assay on HaCaT cells. Principle of this method is to determine the cohesive strength of a keratinocyte monolayer due to stimulation, application of mechanical stress and quantification of resulting fragments (33). Again, the secretion of IL-1 was determined by ELISA. To block inflammasome-mediated caspase activation, the pan-caspase-inhibitor zVad-fmk was added prior to treatment with UVA and antibody.

As expected, treatment with the specific anti-Dsg3 IgG AK23 led to fragmentation of the cell monolayer as a surrogate parameter of acantholysis. The previous irradiation with UVA (subpathogenic level) significantly increased the number of fragments (**Figure 1B**). This effect was reversed by treating cells with the pan-caspase inhibitor zVAD-fmk prior to UVA-irradiation and treatment with anti-Dsg3 antibody, verifying the involvement of caspase activation. In the dissociation assay, additional UVA treatment significantly enhanced the secretion of IL-1α and IL-1β compared to the untreated control condition. This effect was less pronounced in costimulation with AK23 but was reversed by pretreatment with pan-caspase inhibitor zVAD-fmk.

### Innate immune system activation enhances PV-IgG mediated blister formation *ex vivo*

To mimic a more physiological condition, skin explants taken from the safety margins of tumor operations were treated with UVA (15 J/cm²) and subsequently anti-Dsg3 antibody AK23 for 12 hours. As previously shown, the treatment with PV-IgG led to suprabasal acantholysis (34). Here, we titrated the concentration of the anti-Dsg3-antibody AK23 to a subpathogenic concentration, which alone did not induce acantholysis. After irradiation with a non-toxic dose of UVA (35), the threshold to induce acantholysis by anti-Dsg3 IgG was lowered and suprabasal blister formation was observed histologically (**Figure 1C**). UVA irradiation alone did not result in morphological alterations.

## Discussion and perspectives

**Figure 2:**
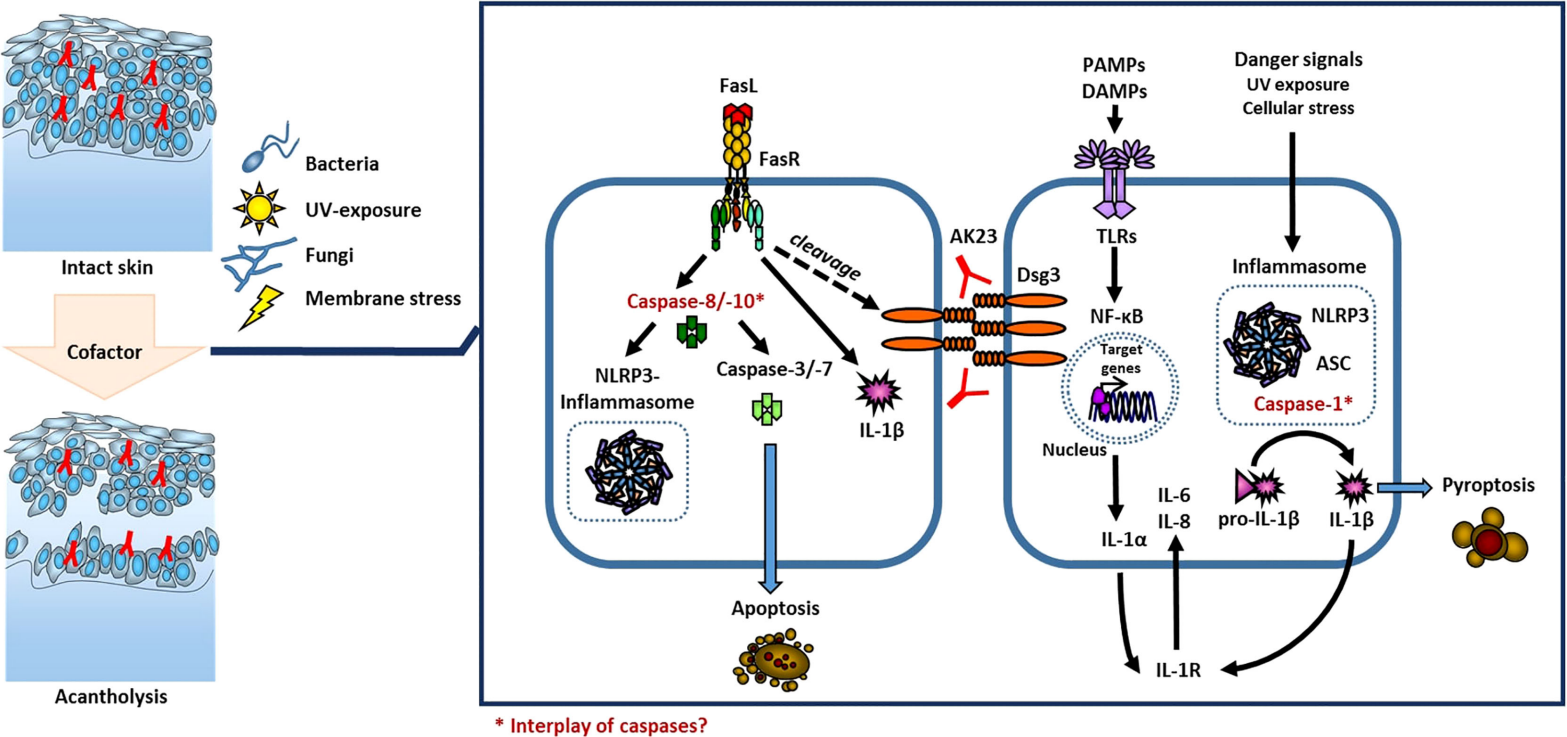
Model of cofactor influenced acantholysis in PV. The local activation of the innate immune system through activation of TLRs and inflammasomes or *via* activation of caspases is known in keratinocytes.

PV is considered a paradigm of an IgG autoantibody-triggered autoimmune disease of the skin which is mainly regulated by cellular components of the adaptive immune system (36). Antigen-specificity and immunological memory make it unique and enable to distinguish from the innate immune system (37). Thus, recent findings reveal that innate immune activation contributes to various autoimmune diseases (38) as e.g. in psoriasis through modulation of T cells (13). The primary immune system as first line of defense against invading danger fulfills immuno-protective functions on the one hand, but once being over-activated is the origin of inflammatory skin diseases. It reacts through the detection of external signals, cytokines, and chemokines, released by inflammatory cells. There are several extra- und intracellular pattern recognition receptors whose activation lead to cytokine release, with Nod-like receptors (NLR) being one of them (39). NLRs upon activation form inflammasomes, intracellular multi-protein complexes which result in caspase-activation. Caspases are proteases involved in cell death and inflammatory responses. Activation of caspase-1 through inflammasome formation leads to pyroptosis (a pro-inflammatory form of cell death) and cleavage of inactive pro-IL-1β to biologically active IL-1β, leading to its secretion (37) (**Figure 2**). In addition, caspase-1 independent mechanisms of IL-1 release were identified, too (40, 41).

To understand the pathogenesis of autoimmune blistering dermatoses and specifically PV, the role of the immune system in the loss of self-tolerance and initiation of autoantibody production (38), and a local influence of the antibodies on the epidermis, finally resulting in acantholysis, have to be clearly distinguished. Aim of the study was to focus on factors needed for blister induction upon antibody binding to the epidermis.

It is known that incubation with anti-Dsg3 IgG can induce acantholysis both *in vitro* and *in vivo* (42, 43). Our results show that the threshold of acantholysis can be lowered by an exogenous cofactor, which in the case of UVA acts *via* activation of the innate immune system. Previous hints for a possible pathophysiological involvement were obtained by detecting elevated levels of innate cytokines from the blister fluid and sera of PV patients (9, 10, 44). We propose local activation of the innate immune system as a possible cofactor in autoantibody-induced acantholysis in the pathogenesis of PV. Specifically, we identified UVA to be a caspase-dependent pathogenic factor.

UVA irradiation of keratinocytes leads to innate immune activation and the secretion of IL-1 and IL-6, important cytokines of the innate immune system (45).

Innate cytokines are known to negatively impact autoimmune diseases (e.g. lupus erythematosus) (46) and were further described as a trigger in the manifestation of pemphigus foliaceus (47). Increased secretion of proinflammatory cytokines upon UV treatment in PV patients has been described previously (48). In our case, the treatment with IgG purified from PV patient sera led to notable changes in expression of the above-mentioned cytokines. Furthermore, UVA irradiation induced the secretion of IL-1α, IL-1β, IL-6 and IL-8 (**Figure 1A**).

Interestingly, keratinocyte monolayers treated with UVA dissociate in significantly more fragments upon mechanical stress in the presence of anti-Dsg3 IgG compared to monolayers treated with anti-Dsg3 IgG only. We are proposing UVA irradiation with subsequent activation of the innate immune system as a cofactor inducing acantholysis after antibody binding *in vitro*. These results were confirmed by an *ex vivo* human skin explant model. Here, UVA also decreased the antibody threshold for anti-Dsg3-mediated acantholysis as the combination of PV antibody and UVA led to acantholysis at a lower antibody concentration compared to untreated skin explants.

Although, UVA cannot entirely explain the occurrence of cutaneous symptoms at the predilection sites (8) as oral mucosa is not exposed to the sun, we detected an obvious impact on acantholysis under experimental conditions. Other exogenous factors related to the activation of the innate immune system may also be pathogenically relevant and should be investigated in further experiments.

Muller et al. argued that the induction of cytokine production by keratinocytes does not contribute to the pathogenesis of PV but are rather induced *via* a secondary activation of the innate immune system by a disturbed epidermal barrier (49). However, as the level of innate cytokines in the serum as well as in the blister fluid of PV patients obviously reflects the dynamics of the disease process and we observed that UV radiation induced cytokine secretion and enhanced autoantibody-mediated acantholysis, these data indicate that cytokines contribute to diseases activation. Additionally, in clinical remission, the levels of innate cytokines decrease, while the amount of endogenous IL-1 receptor antagonist increases (44). Furthermore, stimulation of PBMC in patients with endemic pemphigus foliaceus resulted in higher IL-1β secretion compared to healthy donors (50). These clinical observations have been supported by experimental data showing that IL-1 receptor (IL-1R) knockout mice were protected from blister formation after the injection of PV serum. While injection of pathogenic IgG autoantibodies against Dsgs into newborn mice induced intraepidermal loss of adhesion, this phenotype is absent in mice deficient for IL-1R type 1, the receptor for IL-1α and IL-1β (10).

The functional network of different caspases and cytokines is closely interwoven, and its complexity is yet poorly understood. Whether IL-1 is the cause or the consequence of increased acantholysis remains cryptic and needs to be addressed in future studies. Based on our findings, we propose an additive effect through activation of the innate immune system on acantholysis. Importantly, under experimental conditions, we characterized the UVA-mediated effect being caspase-dependent because the pan-caspase-inhibitor zVAD-fmk was effective to block the effect of UVA on acantholysis. This is interesting because in previous studies in the same cell line zVAD-fmk did not reduce loss of cell adhesion when PV-IgG was applied in the absence of UVA (51). Thus, the additional effect of UVA may explain why in several mouse models a contribution of caspase signaling to acantholysis was observed (52, 53). Furthermore, desmosomes themselves could also play a role in local modulation of the innate immune system (49).

## Concluding remarks

In future studies, the role of the innate immune system as a cofactor in the pathogenesis of PV and the influence of cytokines on desmosomes should be investigated further. Besides the direct effects of innate cytokines, the impact of caspases needs to be elucidated. All in all, we are the first to introduce the local activation of the innate immune system *via* inflammatory caspases as a possible cofactor in the pathogenesis of auto-antibody-induced acantholysis in PV, which might act as starting point for further studies on disease manifestation of autoantibody-mediated diseases.

## Data availability statement

The original contributions presented in the study are included in the article/**supplementary material**. Further inquiries can be directed to the corresponding author.

## Ethics statement

The studies involving human participants were reviewed and approved by the ethical approval: 547/2011BO2, University of Tuebingen, Germany. The patients/participants provided their written informed consent to participate in this study.

## Author contributions

RE, MS, and AY contributed to the conception and design of the study, supported by MH, JW, and JMB. RE, MS, and EW performed the experiments. RE, MS, and AY wrote the manuscript. All authors contributed to the manuscript revision, read, and approved the final version.

## Funding

This work is funded by the grant from the DFG (Deutsche Forschungsgemeinschaft) to the Research Unit FOR 2497 PEGASUS (to AY and JB (both TP3), JW (TP4) and MH (CP and TP8)).

## Conflict of interest

The authors declare that the research was conducted in the absence of any commercial or financial relationships that could be construed as a potential conflict of interest.

## Publisher’s note

All claims expressed in this article are solely those of the authors and do not necessarily represent those of their affiliated organizations, or those of the publisher, the editors and the reviewers. Any product that may be evaluated in this article, or claim that may be made by its manufacturer, is not guaranteed or endorsed by the publisher.

## Supplementary material

Supplementary Figure 1 HaCaT cells were irradiated with the indicated intensity of UVA. After an incubation period of four hours, the expression of IL1A and IL1B was measured by rtPCR.

## Abbreviations

AP-1, Activator Protein 1; BP, Bullous Pemphigoid; Dsg1, Desmoglein 1; Dsg3, Desmoglein 3; ELISA, Enzyme-linked Immunosorbent Assay; FasL, Fas-Ligand; IgG, Immunoglobulin; IL, Interleukin; IL-1R, Interleukin-1 Receptor; PF, Pemphigus foliaceus; PV, Pemphigus vulgaris; rtPCR, real-time PCR; UVA, Ultraviolet A.

## References

[1] Kasperkiewicz M Ellebrecht CT Takahashi H Yamagami J Zillikens D Payne AS. Pemphigus. Nat Rev Dis Primers (2017) 3:17026. doi: 10.1038/nrdp.2017.26 28492232 PMC5901732

[2] Pollmann R Schmidt T Eming R Hertl M. Pemphigus: a comprehensive review on pathogenesis, clinical presentation and novel therapeutic approaches. Clin Rev Allergy Immunol (2018) 54(1):1–25. doi: 10.1007/s12016-017-8662-z 29313220

[3] Didona D Maglie R Eming R Hertl M. Pemphigus: current and future therapeutic strategies. Front Immunol (2019) 10:1418. doi: 10.3389/fimmu.2019.01418 31293582 PMC6603181

[4] Schmitt T Waschke J. Autoantibody-specific signalling in pemphigus. Front Med (Lausanne) (2021) 8:701809. doi: 10.3389/fmed.2021.701809 34434944 PMC8381052

[5] Hertl M Jedlickova H Karpati S Marinovic B Uzun S Yayli S. Pemphigus. S2 guideline for diagnosis and treatment–guided by the European dermatology forum (EDF) in cooperation with the European academy of dermatology and venereology (EADV). J Eur Acad Dermatol Venereol (2015) 29(3):405–14. doi: 10.1111/jdv.12772 25338479

[6] Hsu DY Brieva J Sinha AA Langan SM Silverberg JI. Comorbidities and inpatient mortality for pemphigus in the U.S.A. Br J Dermatol (2016) 174(6):1290–8. doi: 10.1111/bjd.14463 26864457

[7] Kridin K Sagi SZ Bergman R. Mortality and cause of death in patients with pemphigus. Acta Derm Venereol (2017) 97(5):607–11. doi: 10.2340/00015555-2611 28093595

[8] Kneisel A Hertl M. Autoimmune bullous skin diseases. part 1: Clinical manifestations. J Dtsch Dermatol Ges (2011) 9(10):844–56. doi: 10.1111/j.1610-0387.2011.07793.x 21955378

[9] Grando SA Glukhenky BT Drannik GN Epshtein EV Kostromin AP Korostash TA. Mediators of inflammation in blister fluids from patients with pemphigus vulgaris and bullous pemphigoid. Arch Dermatol (1989) 125(7):925–30. doi: 10.1001/archderm.1989.01670190059006 2662908

[10] Feliciani C Toto P Amerio P Pour SM Coscione G Shivji G. *In vitro* and *in vivo* expression of interleukin-1alpha and tumor necrosis factor-alpha mRNA in pemphigus vulgaris: interleukin-1alpha and tumor necrosis factor-alpha are involved in acantholysis. J Invest Dermatol (2000) 114(1):71–7. doi: 10.1046/j.1523-1747.2000.00835.x 10620118

[11] Bhol KC Rojas AI Khan IU Ahmed AR. Presence of interleukin 10 in the serum and blister fluid of patients with pemphigus vulgaris and pemphigoid. Cytokine (2000) 12(7):1076–83. doi: 10.1006/cyto.1999.0642 10880254

[12] Feliciani C Toto P Amerio P. *In vitro* C3 mRNA expression in pemphigus vulgaris: complement activation is increased by IL-1alpha and TNF-alpha. J Cutan Med Surg (1999) 3(3):140–4. doi: 10.1177/120347549900300306 10082594

[13] Ghoreschi K Thomas P Breit S Dugas M Mailhammer R van Eden W. Interleukin-4 therapy of psoriasis induces Th2 responses and improves human autoimmune disease. Nat Med (2003) 9(1):40–6. doi: 10.1038/nm804 12461524

[14] Stern JN Keskin DB Barteneva N Zuniga J Yunis EJ Ahmed AR. Possible role of natural killer cells in pemphigus vulgaris - preliminary observations. Clin Exp Immunol (2008) 152(3):472–81. doi: 10.1111/j.1365-2249.2008.03638.x PMC2453198 18373702

[15] Mantovani A Dinarello CA Molgora M Garlanda C. Interleukin-1 and related cytokines in the regulation of inflammation and immunity. Immunity (2019) 50(4):778–95. doi: 10.1016/j.immuni.2019.03.012 PMC7174020 30995499

[16] Chung Y Chang SH Martinez GJ Yang XO Nurieva R Kang HS. Critical regulation of early Th17 cell differentiation by interleukin-1 signaling. Immunity (2009) 30(4):576–87. doi: 10.1016/j.immuni.2009.02.007 PMC2705871 19362022

[17] Korman N. . doi: 10.1016/s0190-9622(87)70115-7

[18] Kasperkiewicz M Zillikens D. The pathophysiology of bullous pemphigoid. Clin Rev Allergy Immunol (2007) 33(1-2):67–77. doi: 10.1007/s12016-007-0030-y 18094948

[19] Budinger L Borradori L Yee C Eming R Ferencik S Grosse-Wilde H. Identification and characterization of autoreactive T cell responses to bullous pemphigoid antigen 2 in patients and healthy controls. J Clin Invest (1998) 102(12):2082–9. doi: 10.1172/JCI3335 PMC509162 9854043

[20] Thoma-Uszynski S Uter W Schwietzke S Schuler G Borradori L Hertl M. Autoreactive T and b cells from bullous pemphigoid (BP) patients recognize epitopes clustered in distinct regions of BP180 and BP230. J Immunol (2006) 176(3):2015–23. doi: 10.4049/jimmunol.176.3.2015 16424234

[21] Gammon WR Merritt CC Lewis DM Sams WM Jr. Wheeler CE Jr. Carlo JR. Functional evidence for complement-activating immune complexes in the skin of patients with bullous pemphigoid. J Invest Dermatol (1982) 78(1):52–7. doi: 10.1111/1523-1747.ep12497912 7033396

[22] Jordon RE Kawana S Fritz KA. Immunopathologic mechanisms in pemphigus and bullous pemphigoid. J Invest Dermatol (1985) 85(1 Suppl):72s–8s. doi: 10.1111/1523-1747.ep12275497 3891883

[23] Liu Z Giudice GJ Swartz SJ Fairley JA Till GO Troy JL. The role of complement in experimental bullous pemphigoid. J Clin Invest (1995) 95(4):1539–44. doi: 10.1172/JCI117826 PMC295637 7706459

[24] Kylmaniemi M Autio P Oikarinen A. Interleukin 1 alpha (IL-1 alpha) in human skin *in vivo*: lack of correlation to markers of collagen metabolism. Acta Derm Venereol (1994) 74(5):364–7. doi: 10.2340/0001555574364367 7817673

[25] Schmidt E Mittnacht A Schomig H Dummer R Brocker EB Zillikens D. Detection of IL-1 alpha, IL-1 beta and IL-1 receptor antagonist in blister fluid of bullous pemphigoid. J Dermatol Sci (1996) 11(2):142–7. doi: 10.1016/0923-1811(95)00435-1 8869035

[26] Schmidt E Bastian B Dummer R Tony HP Brocker EB Zillikens D. Detection of elevated levels of IL-4, IL-6, and IL-10 in blister fluid of bullous pemphigoid. Arch Dermatol Res (1996) 288(7):353–7. doi: 10.1007/BF02507102 8818181

[27] Fang H Li Q Wang G. The role of T cells in pemphigus vulgaris and bullous pemphigoid. Autoimmun Rev (2020) 19(11):102661. doi: 10.1016/j.autrev.2020.102661 32942041

[28] Das D Akhtar S Kurra S Gupta S Sharma A. Emerging role of immune cell network in autoimmune skin disorders: An update on pemphigus, vitiligo and psoriasis. Cytokine Growth Factor Rev (2019) 45:35–44. doi: 10.1016/j.cytogfr.2019.01.001 30773437

[29] Tavakolpour S Mahmoudi H Mirzazadeh A Balighi K Darabi-Monadi S Hatami S. Pathogenic and protective roles of cytokines in pemphigus: A systematic review. Cytokine (2020) 129:155026. doi: 10.1016/j.cyto.2020.155026 32058276

[30] Rox JM Reinartz J Kramer MD. Interleukin-1 beta upregulates tissue-type plasminogen activator in a keratinocyte cell line (HaCaT). Arch Dermatol Res (1996) 288(9):554–8. doi: 10.1007/BF02505254 8874752

[31] Schaefer BM Jaeger CJ Kramer MD. Plasminogen activator system in pemphigus vulgaris. Br J Dermatol (1996) 135(5):726–32. doi: 10.1111/j.1365-2133.1996.tb03881.x 8977672

[32] Radeva MY Walter E Stach RA Yazdi AS Schlegel N Sarig O. ST18 enhances PV-IgG-Induced loss of keratinocyte cohesion in parallel to increased ERK activation. Front Immunol (2019) 10:770. doi: 10.3389/fimmu.2019.00770 31057535 PMC6478701

[33] Caldelari R de Bruin A Baumann D Suter MM Bierkamp C Balmer V. A central role for the armadillo protein plakoglobin in the autoimmune disease pemphigus vulgaris. J Cell Biol (2001) 153(4):823–34. doi: 10.1083/jcb.153.4.823 PMC2192383 11352942

[34] van der Wier G Pas HH Jonkman MF. Experimental human cell and tissue models of pemphigus. Dermatol Res Pract (2010) 2010:143871. doi: 10.1155/2010/143871 20585596 PMC2877615

[35] Evans-Johnson JA Garlick JA Johnson EJ Wang XD Oliver Chen CY. A pilot study of the photoprotective effect of almond phytochemicals in a 3D human skin equivalent. J Photochem Photobiol B (2013) 126:17–25. doi: 10.1016/j.jphotobiol.2013.07.006 23892186

[36] Janeway CA Jr Travers P Walport M Shlomchik Mark J. Immunobiology: The immune system in health and disease. 5th edition. New York: Garland Science (2001).

[37] Gasteiger G D'Osualdo A Schubert DA Weber A Bruscia EM Hartl D. Cellular innate immunity: an old game with new players. J Innate Immun (2017) 9(2):111–25. doi: 10.1159/000453397 PMC6738785 28006777

[38] Saferding V Bluml S. Innate immunity as the trigger of systemic autoimmune diseases. J Autoimmun (2020) 110:102382. doi: 10.1016/j.jaut.2019.102382 31883831

[39] Alberts B Johnson A Lewis J Raff M Roberts K Walter P. Molecular Biology of the Cell. 4th edition. New York: Garland Science (2002).

[40] Karmakar M Sun Y Hise AG Rietsch A Pearlman E. Cutting edge: IL-1beta processing during pseudomonas aeruginosa infection is mediated by neutrophil serine proteases and is independent of Nlrc4 and caspase-1. J Immunol (2012) 189(9):4231–5. doi: 10.4049/jimmunol.1201447 PMC3482477 23024281

[41] Kono H Orlowski GM Patel Z Rock KL. The IL-1-dependent sterile inflammatory response has a substantial caspase-1-independent component that requires cathepsin c. J Immunol (2012) 189(7):3734–40. doi: 10.4049/jimmunol.1200136 PMC3448805 22914048

[42] Amagai M Karpati S Prussick R Klaus-Kovtun V Stanley JR. Autoantibodies against the amino-terminal cadherin-like binding domain of pemphigus vulgaris antigen are pathogenic. J Clin Invest (1992) 90(3):919–26. doi: 10.1172/JCI115968 PMC329947 1522242

[43] Sanchez-Carpintero I Espana A Pelacho B Lopez Moratalla N Rubenstein DS Diaz LA. *In vivo* blockade of pemphigus vulgaris acantholysis by inhibition of intracellular signal transduction cascades. Br J Dermatol (2004) 151(3):565–70. doi: 10.1111/j.1365-2133.2004.06147.x 15377341

[44] Bhol KC Desai A Kumari S Colon JE Ahmed AR. Pemphigus vulgaris: the role of IL-1 and IL-1 receptor antagonist in pathogenesis and effects of intravenous immunoglobulin on their production. Clin Immunol (2001) 100(2):172–80. doi: 10.1006/clim.2001.5061 11465946

[45] Kondo S. The roles of keratinocyte-derived cytokines in the epidermis and their possible responses to UVA-irradiation. J Investig Dermatol Symp Proc (1999) 4(2):177–83. doi: 10.1038/sj.jidsp.5640205 10536996

[46] Lehmann P Holzle E Kind P Goerz G Plewig G. Experimental reproduction of skin lesions in lupus erythematosus by UVA and UVB radiation. J Am Acad Dermatol (1990) 22(2 Pt 1):181–7. doi: 10.1016/0190-9622(90)70020-i 2179293

[47] Igawa K Matsunaga T Nishioka K. Involvement of UV-irradiation in pemphigus foliaceus. J Eur Acad Dermatol Venereol (2004) 18(2):216–7. doi: 10.1111/j.1468-3083.2004.00900.x 15009310

[48] Li HY Zhang FR Deng DQ. Relationship between UV-irradiated HaCaT cell cytokines and Th1/Th2 imbalance. Genet Mol Res (2015) 14(3):7976–85. doi: 10.4238/2015.July.17.5 26214479

[49] Muller L Hatzfeld M Keil R. Desmosomes as signaling hubs in the regulation of cell behavior. Front Cell Dev Biol (2021) 9:745670. doi: 10.3389/fcell.2021.745670 34631720 PMC8495202

[50] Rocha-Rodrigues DB Paschoini G Pereira SA dos Reis MA Teixeira Vde P Rodrigues Junior V. High levels of interleukin-1 in patients with endemic pemphigus foliaceus. Clin Diagn Lab Immunol (2003) 10(5):741–3. doi: 10.1128/cdli.10.5.741-743.2003 PMC193914 12965897

[51] Schmidt E Gutberlet J Siegmund D Berg D Wajant H Waschke J. Apoptosis is not required for acantholysis in pemphigus vulgaris. Am J Physiol Cell Physiol (2009) 296(1):C162–72. doi: 10.1152/ajpcell.00161.2008 18987254

[52] Schmidt E Waschke J. Apoptosis in pemphigus. Autoimmun Rev (2009) 8(7):533–7. doi: 10.1016/j.autrev.2009.01.011 19189866

[53] Lotti R Shu E Petrachi T Marconi A Palazzo E Quadri M. Soluble fas ligand is essential for blister formation in pemphigus. Front Immunol (2018) 9:370. doi: 10.3389/fimmu.2018.00370 29535737 PMC5834757

