# Supplementary figures and images for: Innate immune activation as cofactor in pemphigus disease manifestation
Source: Front Immunol. 2022 Jul 19;13:898819. doi: 10.3389/fimmu.2022.898819 (PMC9343989; doi:10.3389/fimmu.2022.898819)

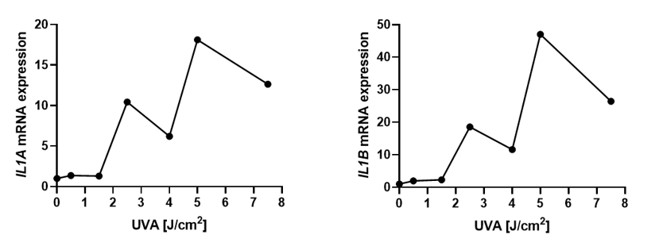

Supplement: Supplementary Figure 1 — HaCaT cells were irradiated with the indicated intensity of UVA. After an incubation period of four hours, the expression of IL1A and IL1B was measured by rtPCR. [file Image_1.jpeg]
